# Adoption of a deep learning-based neural network model in the psychological behavior analysis of resident tourism consumption

**DOI:** 10.3389/fpubh.2022.995828

**Published:** 2022-12-19

**Authors:** Zicong Ye, Xiantao Huang

**Affiliations:** School of Physical Education and Sport (College of Evergrande Football), Wuhan University of Science and Technology, Wuhan, China

**Keywords:** consumer psychology analysis, convolutional neural network, human behavior recognition, target detection, tourism consumption, leisure sports

## Abstract

With the development of society and the continuous progress of science and technology, it has become the mainstream measure to promote the development of the social economy through science and technology. Therefore, to improve the current situation of tourism consumption, improve the consumer sentiment of tourists, and promote the development of the tourism economy, the convolutional neural network (CNN) technology model is used to analyze the tourist's consumer psychology and behavior. Based on this, the user's consumption situation is analyzed, thus providing support for the intelligent improvement of tourism consumption. First, the basic characteristics of tourism consumption mood and behavior are introduced, and the methods to improve the tourism consumption mood and behavior are briefly introduced. Then, the CNN algorithm is employed to identify consumers' travel consumption behaviors and emotions. To improve the recognition effect, the algorithm is combined with skeleton node behavior recognition and video image behavior recognition. Finally, the performance of the designed algorithm is tested. The accuracy of the human behavior recognition (HBR) algorithm is more than 0.88. Compared with the detection effect of the HBR algorithm, the combined algorithm adopted in this work can reduce the image processing time and improve the detection efficiency. The multithread method can effectively reduce the complexity of the model and improve the recognition accuracy. The test results on different data sets show that the proposed algorithm can better adapt to the changes in identification samples and obtain more accurate recognition results compared with similar algorithms. In summary, this study not only provides technical support for the rational analysis of consumer sentiment and consumer behavior but also contributes to the comprehensive development of the tourism market.

## 1. Introduction

With the rapid development of the economy, the per capita income of residents in China continues to increase, and the requirements for living standards are gradually increasing. Therefore, an increasing number of people use tourism as a daily leisure method. At present, the Chinese tourism market is still in the early stage compared to other tourism markets, and China is expected to become the largest domestic tourism market, making the development prospects of the Chinese tourism market very promising. The key to winning the competition in the Chinese and even the global tourism markets is for companies in tourist destinations to formulate their development strategies based on the travel trends of Chinese tourists and the psychology of consumers. Tourism consumption patterns are the general laws and general characteristics of consumption behaviors of tourists in a certain tourism consumption period, reflecting the consumption patterns and characteristics of individuals and groups. Therefore, it is possible to promote tourism consumption by analyzing the psychological needs and behaviors of tourists (1).

The tourism consumption behavior of tourists is a variety of actions for obtaining, using, and disposing of consumer goods or services under their psychological drive, including the process of tourists making decisions about these actions. With the development of shopping markets in tourist destinations, the shopping behaviors of consumers mainly occur in large supermarkets, such as duty-free areas in airports or stations. The shopping mall can analyze the consumption psychology and intention of tourists according to the consumption ability and shopping behavior of tourists and then use the shopping guide robot of the shopping mall to guide users in shopping behaviors with corresponding economic ability to provide help for the economic income of tourist destinations and shopping mall planning (2). With the development of computer technology, shopping-guide robots have been applied widely in shopping malls.

In this work, a tourist consumption psychology and behavior analysis model based on a convolutional neural network (CNN) is proposed and applied to an intelligent shopping guide robot in a shopping mall. Robots are used to identify the shopping behaviors of consumers in shopping malls in real time, determine the type of consumption of tourists, and infer their consumption psychology. In this way, consumers are guided to consume in areas with obvious shopping intentions, which can help the economic benefits of tourist destinations. The research innovation of this work is to help the shopping guide robot to quickly judge the consumer psychology and consumption type by establishing the relationship model between tourism consumption psychology and actual behaviors of residents and establishing a real-time behavior analysis algorithm. In addition, personalized consumption services are provided to enhance consumers' travel and shopping experience as well as local tourism economic income according to their consumption characteristics.

In summary, this study aims to deeply explore consumer behavior and consumer psychology and then provide a reference for improving consumer behavior. This work has four parts. First, by referring to the relevant literature, the research status of the relevant research field is analyzed. Second, consumer behavior and consumer psychology are combined, and the corresponding psychological model is established. Then, the experimental model is designed and trained by a neural network algorithm. Finally, the ability of the designed model to deal with the corresponding problems is verified by experiments, and the shortcomings of this work are summarized. This study not only provides technical support for improving consumer sentiment but also contributes to promoting social and economic development.

## 2. Recent related works

Research on interactive shopping behavior has been widespread. Prabakaran et al. (3) developed an automatic human-computer interaction shopping cart. By installing intelligent mobile robots on the shopping cart, the webcam was used to identify specific marks on customers, guide them to the planned purchase location, and record the purchase on the shopping list. Garcês et al. (4) pointed out that when tourists purchase the core product, tourism service, they will also be influenced by the publicity and promotion of its extension product, namely, the tourism product. Moreover, the researcher pointed out that the study of consumer psychology can not only understand the current consumption tendency and purchasing behavior of tourists but also predict their future consumption tendency. A good grasp of the relationship between consumer psychology and marketing countermeasures will give tourism enterprises more competitive advantages (4). Islam et al. (5) developed an intelligent shopping robot that provides corresponding shopping services by observing the head direction, body direction, and shopping behavior of customers. According to the video, the robot uses the deep neural network (DNN)-based human posture estimator to extract the skeleton of 18 joints of the human body and builds a database based on the information obtained. Using the developed gated recurrent neural network topology, the collected information was classified to obtain different motion states (for example, reaching the shelf, checking the goods, and pulling the hand from the shelf). Experimental results showed that the overall accuracy of the designed recognition model reached 82% (5). Therefore, consumers' shopping intentions can be understood by identifying and analyzing their behaviors or shopping goals.

Some researchers have studied how visitors' emotions and mental states can be judged by their behavior. González-Rodríguez et al. (6) analyzed the emotions of tourists using data recorded by a facial expression recognition software application. The tourists were then asked to rate the tour guide's performance. Second, the structural equation model was used to verify the strong correlation between emotion and satisfaction. The output of the model provided a potential method to measure the satisfaction of tourism customers. The results showed that the information obtained by facial expression recognition was consistent with the customer satisfaction results obtained by the questionnaire survey. It was mainly based on the use of facial expression recognition software and the measurement of internal emotions in specific tourism scenes (6). Liu (7) established a precision marketing model *via* neural networks and studied two factors that have a significant impact on marketing revenue (user loss and user value enhancement). Combined with the characteristics of user groups and the application market of products, the model proposes the corresponding precision marketing strategy. Experimental results showed that the big data information platform can increase users' sensitive information for precision marketing (7). Peng (8) studied a sentiment analysis model based on text mining technology to analyze users' emotions by processing comments, opinions, recommendations, and feedback on the Internet. In addition, the obstacles of accurate meaning analysis of emotion and judgment of emotion polarity have also been discussed in depth (8).

The current research status suggests that through neural networks or data mining, the emotional and psychological information of users can be obtained from the data and then help the machine make the next judgment. To expand the application scenarios of neural network algorithms, it is proposed to further apply them to shopping-guide activities in large shopping malls. The CNN is applied to analyze consumer behavior and psychology to promote the economic development of tourist destinations. The designed model is utilized to classify consumer types by combining consumer psychology-related content, and personalized consumer services are promoted through shopping guide robots in shopping malls to accelerate the development of the local tourism economy.

## 3. Research methodology and research model

### 3.1 Analysis and research on residents' consumption psychology and behavior

The theory of reasoned action (TRA) is based on the behavioral attitudes of individuals and subjective norms to rationally predict individual behavior. The basic assumption of TRA is that people are rational and will consider the meaning and results of behavior by synthesizing various pieces of information when making a certain action (9). Compared with the TRA, the planned TRA not only considers the influence of behavior attitude and subjective norms on behavior intention but also introduces the variable of perceived behavior control. In this theory, behavioral beliefs, normative beliefs, and control beliefs affect the above control variables. Therefore, the stronger the purchase intention of tourists is, the higher the social influence and group recognition, and the stronger the controllability of perceived behavior control is, the higher the success rate of purchase (10).

Tourism motivation is the internal driving force for residents to engage in tourism activities and is influenced by the personal knowledge level, economic income, life experience, and social environment of residents (11). Dynamically speaking, tourism consumption is the behavior of tourists to satisfy their psychological needs after purchasing tourism products and services. Statically speaking, tourism consumption is the value of the products and services generated for tourists. Consumer behavior is the performance of consumer psychology, such as demand psychology, purchase motivation, consumption intention, actual demand, shopping environment, and product evaluation. The main performance of consumer behavior is purchase behavior. The restrictive factors of consumption behavior include the following points (12).

The first point is need, including physical, social, and psychological needs. It is the direct motivation for consumers to purchase.

The second point is the level of disposable income of consumers and the level of commodity prices. Total household consumption is related to the level of disposable income. The increase in disposable income will promote household consumption to a certain extent.

The third point is the characteristics of goods and subsequent repair and maintenance services. The attributes of goods will affect the purchasing behavior of consumers.

The fourth is the influence of the social environment. The needs of consumers are greatly influenced by social and psychological needs (13).

Therefore, tourists are influenced by multiple factors, such as motivation, personality, price, and service quality, when consumption decisions are made. These factors work together and influence the consumption psychology and behavior of tourists according to certain internal logical relationships. Therefore, the study of consumer behavior refers to analyzing various factors affecting consumer psychology and consumer behavior of different consumers, thereby revealing the changing rules of consumer behavior. Studies on the psychology of consumers include rational behavior theory and planned behavior theory.

### 3.2 Classification of consumer behavior

Consumer behavior consists of two main components. The first refers to the decision-making process of consumers in purchase, which reflects the psychological activities and behavioral tendencies of consumers before they use and dispose of the products and services they buy. It is the formation process of their consumption attitudes. The second refers to the actions of consumers, which is the practice process of purchase decisions in essence. The two parts of consumer behavior interact with each other, forming a complete process of consumption behavior (14). Combined with consumer behavior and consumer psychology, residents' consumers are divided into five types as follows.

The first type is the mental type. When this kind of consumer is buying, they already undertake a meticulous plan and do not want to spend energy trying new brands.

The second type is the thrifty type. These consumers are very sensitive to price and will compare the price, packaging, and weight of similar products when making a purchase. They will think and hesitate for a long time before buying more affordable products.

The third type is brand orientation. Consumers usually have some consumption ability and knowledge level and will choose brand consumption to improve their taste of life and spiritual needs.

The fourth type is impulsive. This type of consumer will show an impulse to buy when they see discount information or new products in the mall.

The fifth group is terminal interceptors. Such consumers usually lack independent opinions and have little understanding of the characteristics of the products they buy. They are easily influenced by promoters in shopping malls and change their previous purchase intentions (15).

To better analyze the consumer psychology and behavior of residents in tourist destinations, it plans to use shopping-guide robots in shopping malls in tourist destinations to track and judge the shopping activities of consumers in real time. The image module of the robot combined with the recognition and analysis algorithm and related indicators (for example: the brand area where the consumer is located, the stay time in the area, the purchase intention, the income level, etc.) are adopted to judge the type of consumer. The shopping-guide robot can provide corresponding consumption guidance according to the types of consumers. To better design the model, the following hypotheses are made on the realization conditions of the designed model.

Hypothesis 1: the application scenario of the designed model is a shopping guide robot in a large shopping mall. Based on the follow-up service of the shopping-guide robot and the collected video image information, the designed model is used to identify the behavior of consumers (16).

Hypothesis 2: in the setting scenario, the results of all consumer behavior classifications correspond to the proposed consumer psychology types, and all consumer behaviors made by consumers represent the process and state of their psychological changes (17).

Hypothesis 3: the designed and trained model can identify and judge the behaviors of consumers in real time and make judgments on the current psychological conditions of the consumers based on the classification results (18).

**Figure 1:**
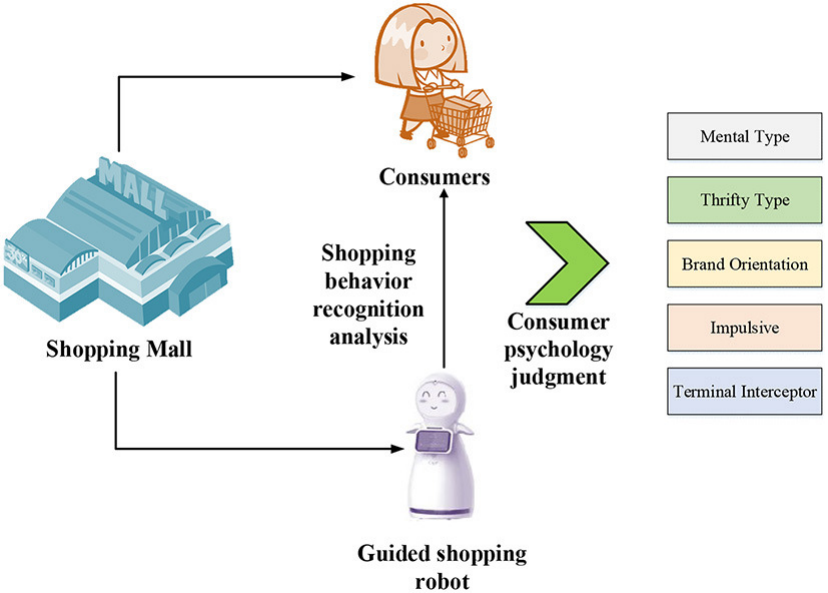
Psychological analysis and type judgment of consumer behavior.

In view of the above research hypotheses and aimed to identify and analyze the behavior of consumers during shopping, an HBR algorithm based on CNN is designed by consulting related literature to identify the behaviors of consumers to determine their willingness to consume and the types of consumers more accurately. Figure 1 is a schematic diagram of consumer behavior recognition and psychological analysis designed.

### 3.3 Behavior analysis algorithm based on CNN

**Figure 2:**
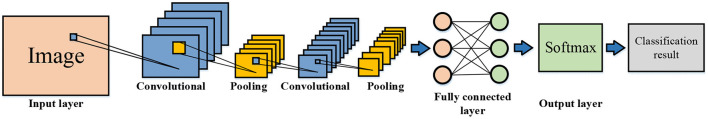
Convolutional neural network structure diagram.

A CNN is a feedforward neural network that includes convolutional calculations and shows a deep structure. It includes five structures: the input layer, convolution layer, pooling layer, fully connected layer, and output layer (as shown in Figure 2). In the schematic diagram of Figure 2, the input layer reads the pixel matrix of the input image and then outputs it to the convolutional layer with multiple feature surfaces, and there are multiple neurons in each feature surface (19). The input of each neuron node is the convolution block of the upper layer network, and the convolution block in the convolution layer can be used to extract the depth information of the image and obtain higher-dimensional feature information. The pooling layer takes dimensionality reduction operations on the high-level features output by the convolutional layer. The neurons between the layers in the fully connected layer are fully connected, and the feature information obtained by the convolutional layer and the pooling layer is classified (20). The output layer uses the softmax function to classify features and output results. After calculation of the probability distribution of each category, the category with the highest probability is the category of the test sample.

Compared with the traditional artificial network, the CNN has the characteristics of local perception and weight sharing. Because the CNN adopts parameter sharing and sparse connection, there are few parameters, and the calculation process is faster. In addition, the CNN has translation invariance. Even if the target position in the image is shifted to another part of the image, the CNN can still identify the target well, and the output result is the same as before the original translation (21). Therefore, a CNN can be used to establish a target detection algorithm with excellent performance.

Behavior recognition refers to recognizing and understanding people's actions in images and expressing them quantitatively (22). Using CNN-based behavior recognition, the characteristic information of the image obtained by the image module of the shopping guide robot is recognized and collected, which is then classified according to the corresponding classification algorithms (23). In view of the complexity of the environment in the shopping mall, two parts are designed based on bone node behavior image recognition and video image behavior recognition to complete the HBR. The image recognition of bone nodes is less affected by ambient light, but the relevance among recognized actions is poor (24). The robustness of video image recognition is poor, but the recognized behavior relevance is good. Therefore, both algorithms are adopted to improve the recognition effect of the robot.

### 3.4 Human skeleton recognition algorithm based on a spatio temporal graph convolutional network (ST-GCN)

The human skeleton recognition algorithm relies on the accurate extraction of human bones. “Openpose” technology can quickly and accurately extract human joint information. Recognition algorithms based on video images adopt video frame sequences for inspection and feature extraction. A behavior of a person is composed of a series of action states (25). The characteristic changes in bone nodes during the process are marked by recognizing the state during the action, and the changes in joints at different time points are related.

Then, the dynamic models of spatial correlation and temporal correlation are established. The behavior is classified and recognized through the analysis of the action characteristics in the two dimensions of time and space (26). The initial motion recognition knowledge expresses the feature vector of the joint coordinates at a certain moment, combined with its time sequence information for analysis, but ignores the spatial characteristics between the joint points (27).

**Figure 3:**
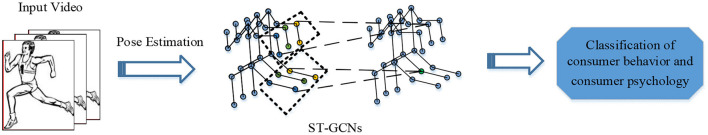
Flow chart of the spatiotemporal graph convolutional network algorithm.

I. Aiming at this, an autonomous learning algorithm is adopted to combine the spatial and temporal feature information of the joints, which is called the spatial temporal graph convolution network (ST-GCN). In the ST-GCN algorithm, graph convolution is applied to the ST-GCN model to realize the description of the skeleton graph sequence (28, 29). Figure 3 shows that each node corresponds to a joint point through the design of the skeleton diagram sequence.

The time dimension and space dimension of bone joints are combined through the connection of the space edges of adjacent joints and the connection of the time edges of the same skeleton node at different times to establish a multilayer ST-GCN (30). After the ST-GCN algorithm is adopted to obtain the bone joint structure and construct the space-time relationship diagram, the action feature information is extracted through multiple spatial-temporal graph convolution processes, and finally, the features are classified to complete the recognition process.

II. The video frame is taken as the input of the video image recognition algorithm, the feature is extracted from the video frame sequence, and features are classified for behavior recognition (31). The commonly used video frame recognition method is the two-stream convolutional network algorithm. The dual-stream CNN utilizes video frames and optical flow field images extracted from them to train two recognition models. After training, the two models containing static information and short time sequence information are fused (32). Optical flow information provides distance information about food through its speed, and optical flow can reflect the angle information. The faster the objects at right angles to the human eye move, the smaller the angle, and the lower the speed of the object's movement.

In the dual-stream CNN architecture, the dense optical flow sequence is obtained by calculating the dense optical flow information between two adjacent video frames in the video frame sequence, which becomes the time domain information. Two CNN models are combined through red–green–blue (RGB) images and dense optical flow information training (33, 34). Then, the information that can extract the time dimension and space dimension in the behavior video is obtained. Finally, the extracted feature information is classified (35).

**Figure 4:**
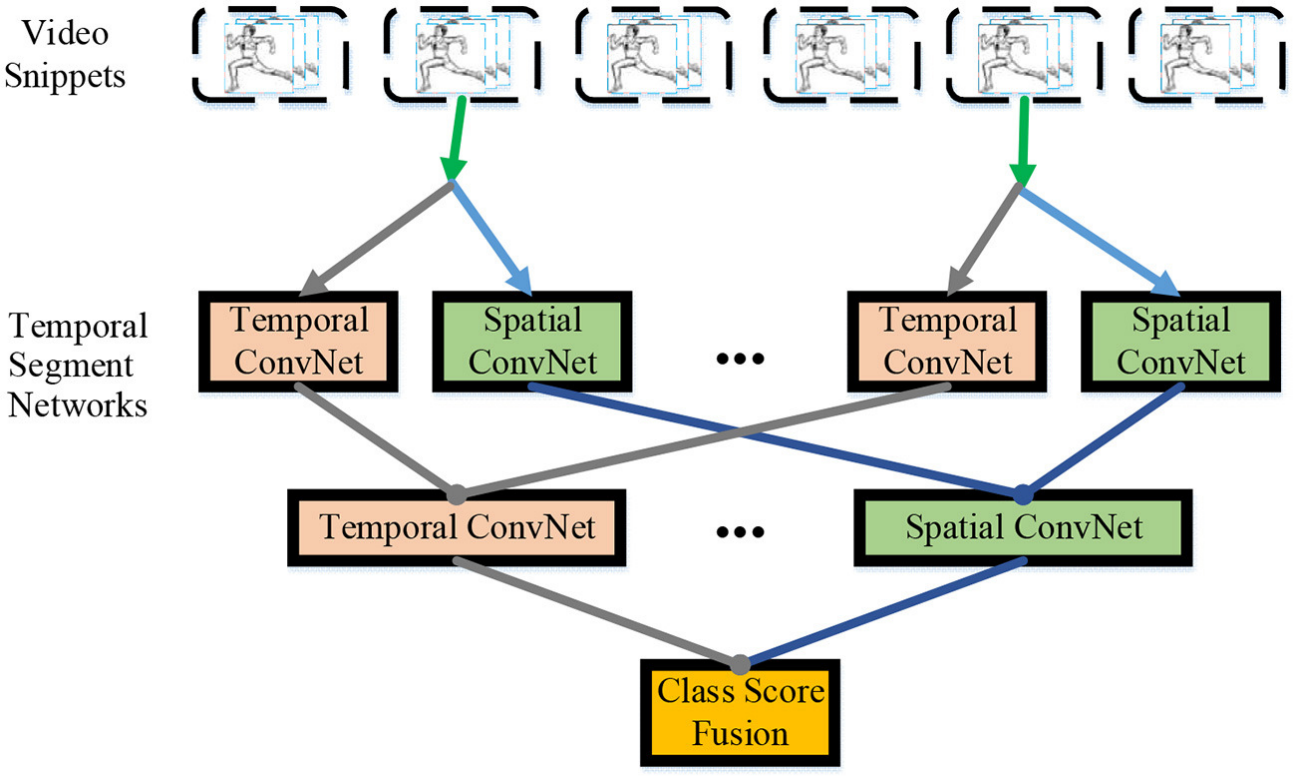
Flowchart of the TSN algorithm.

The algorithm with the best recognition accuracy in the dual-stream CNN algorithm is the temporal segment network (TSN) algorithm. The processing flow of the TSN algorithm is shown in Figure 4. As shown in the figure, the TSN algorithm changes the original RGB image and dense optical flow into four inputs, changes the single dual-stream CNN model to a multisegment dual-stream CNN recognition process, and divides the video into multiple videos and inputs it to the network for inspection (36). In the model recognition, the two algorithms designed will be used to classify and count the behaviors of consumers, and it will correspond to one of the mental models established in the previous article according to the results of the model's classification of behaviors, which is undertaken as a model to identify and evaluate consumer psychology types of consumers.

### 3.5 Experimental design and data set

In this work, data sets are mainly used to study and evaluate the performance of the designed model, and the main plan aims to identify the behavior of consumers carrying or wearing goods during the shopping process to reduce the calculation amount of the shopping guide robot and better judge the psychology of consumers. To better realize the application effect of the target detection algorithm in practice, a training set containing 2,200 images, such as shoes, cups, and hats, is established according to the characteristics of the detection target in the shopping mall. A total of 1,000 images are randomly selected as the training set of the algorithm, and 977 images are randomly selected from the remaining images as the test set (because part of the images in the sample set are obtained through image transformation, such as rotation and flipping, and part of the images are retained as backup to ensure the diversity of the training images) (37). In this study, two experiments are designed to verify the accuracy and time complexity of behavior recognition based on a deep learning algorithm. First, a test set containing 977 human behavior images is used for testing. The images in the test chart contain one or more of the three behaviors. The number of images containing this behavior is counted, and the algorithm is employed to verify the occurrence times of this behavior in the image to obtain the recognition accuracy of this behavior (38). After testing, 977 pictures showed people wearing shoes and hats, and 439 pictures showed people holding cups (people in the pictures were taken in front, side, and back, etc.) (39). Obviously, the experiment in this work is mainly divided into two parts. First, the model designed in this work is used to study and evaluate consumer behavior, and then the model is compared with other advanced models to highlight the characteristics of the model.

The Kinetics-Skeleton data set and ChaLearn Gesture Challenge 2011 (CGD2011) data set are used to better test the performance of the designed algorithm. The Kinetics-Skeleton data set contains 300,000 videos and 400 types of actions, and the videos contain skeleton key point information (40). The CGD2011 data set contains 25,000 gesture images, and each gesture video is 10 frames per second (41). The NTU-RGB + D data set contains three-dimensional skeleton sequences collected with Microsoft Kinect. The data set includes ~60,000 videos and 60 action categories. Table 1 shows the conditions of the equipment used in the experiment (42).

**Table 1:** Device configuration information.

**Parameter**	**Customer**	**Server**
Display routine	Qt 5.7 VS2015	Linux-dash
System	Win7	CentOS 7.3.1611
CPU	i7-7700	Intel Xeon E5-2680
GPU	GeForce GTX 960	GeForce GTX Titan V
The camera	Kinect v1	
Hard drive capacity	16GB+512GB	512GB+4TB

**Figure 5:**
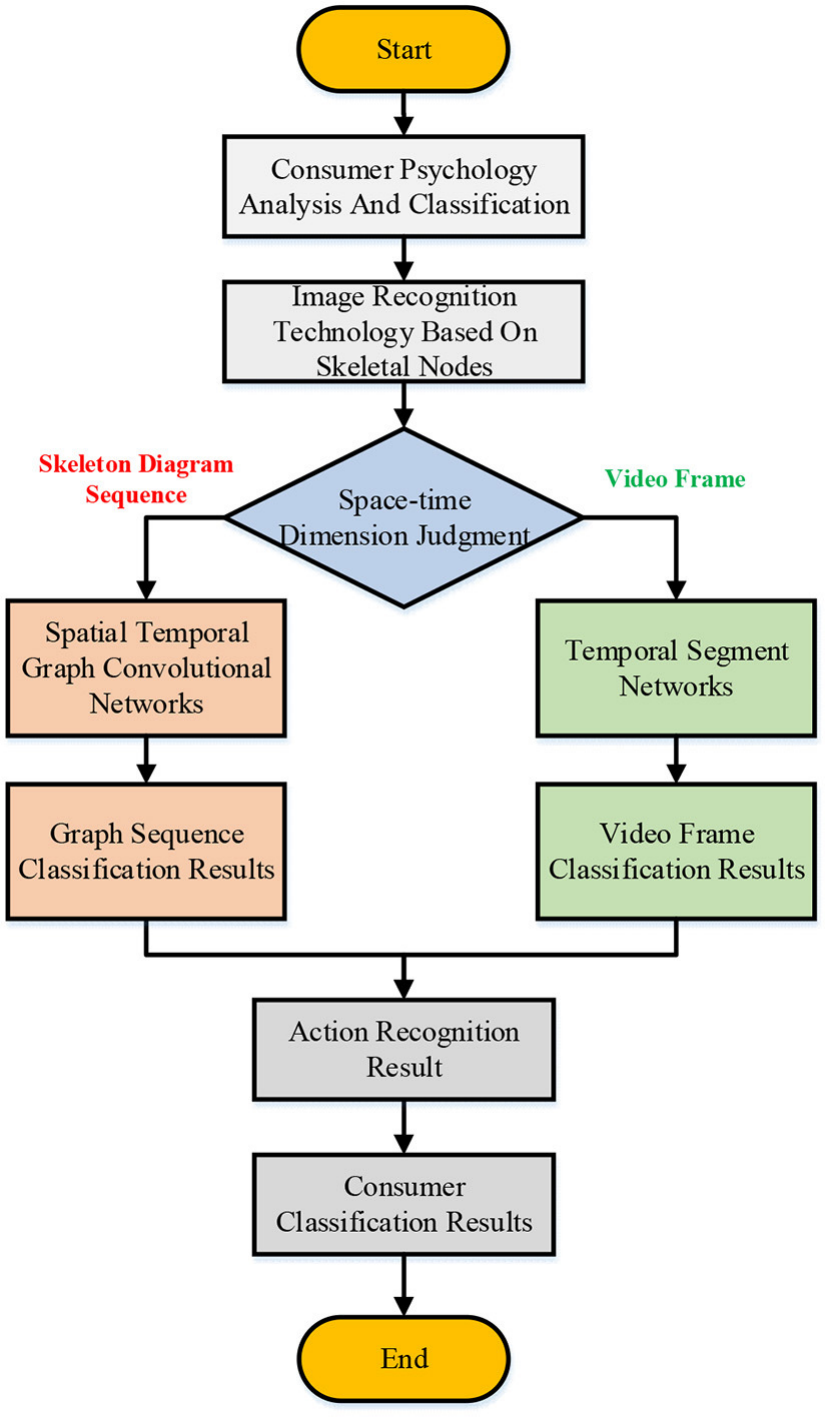
Experiment flows.

Figure 5 is a flowchart of the experiment. It is observed that the image recognition process of the designed model is divided into two parts: skeleton diagram sequence recognition and video frame recognition. Then, the recognition results of the two algorithms are integrated to improve the recognition accuracy of the model. The network parameters of the neural network include the network nodes, initial weights, minimum training rate, dynamic parameters, number of iterations and allowable errors. In this work, the relevant settings of the relevant literature and the method of repeated experiments are combined to determine the optimal network parameters of the model.

In the study, the model will be tested in two parts. The first is to test the effect of model target recognition, and the second is to compare the recognition results of the model and similar algorithms. To compare the classification effect of the classifier, it will be based on the actual performance of the model (positive and negative) and the predicted performance (true and false). The following evaluation indicators are adopted to evaluate the classification effect of the classifier. TP means that a positive sample is classified as a positive sample; FP means that a negative sample is classified as a positive sample; TN refers to a positive sample being classified as a negative sample; and FN refers to a negative sample being classified as a negative sample (positive samples refer to samples that belong to a certain category, and negative samples refer to samples that do not belong to a certain category).

I. Accuracy describes the classification accuracy of the classifier.


(1) $$\begin{array}{l} Accuray=\frac{TP+FN}{TP+FP+TN+FN} \end{array}$$

II. Precision indicates the total proportion of positive samples correctly classified by the classifier.


(2) $$\begin{array}{l} Precision=\frac{TP}{TP+FP} \end{array}$$

## 4. Results and discussion

### 4.1 Model training and testing

**Figure 6:**
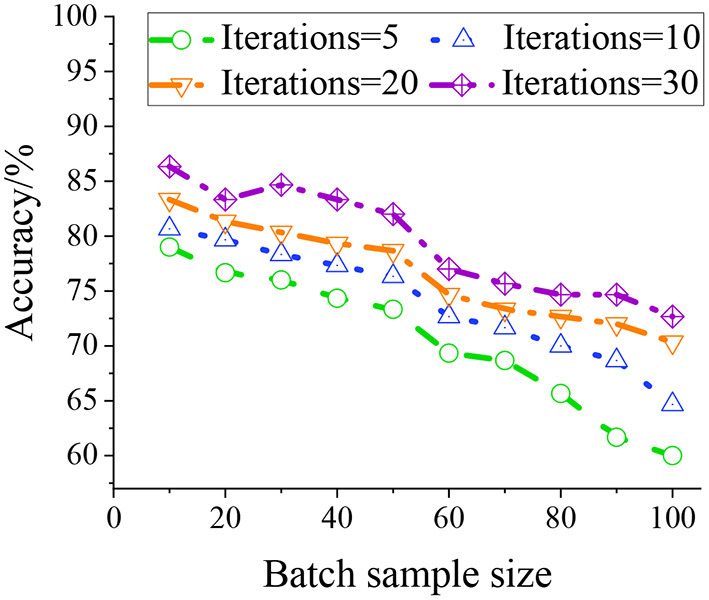
Influence of the number of samples in different batches on the recognition effect.

In network training, the number of batch samples from 1 to 100 is tested with iterations of 5, 10, 20, and 30 to determine the impact of the number of samples in batches on the model recognition effect. The experimental results are shown in Figure 6.

As illustrated in Figure 6, the number of batch samples is negatively correlated with the recognition rate. As the number of batch samples increases, the accuracy rate shows a downward trend but drops significantly when the number of batch samples is 50–60. Therefore, to balance the relationship between the number of batch samples and the number of iterations, the number of batch samples used in the study is 50. With this sample batch, the model can analyze the samples more efficiently, thereby improving the accuracy of the design model prediction. The number of iterations of the model will affect the recognition effect. The figure reveals that the more iterations there are, the higher the recognition accuracy of the model.

**Figure 7:**
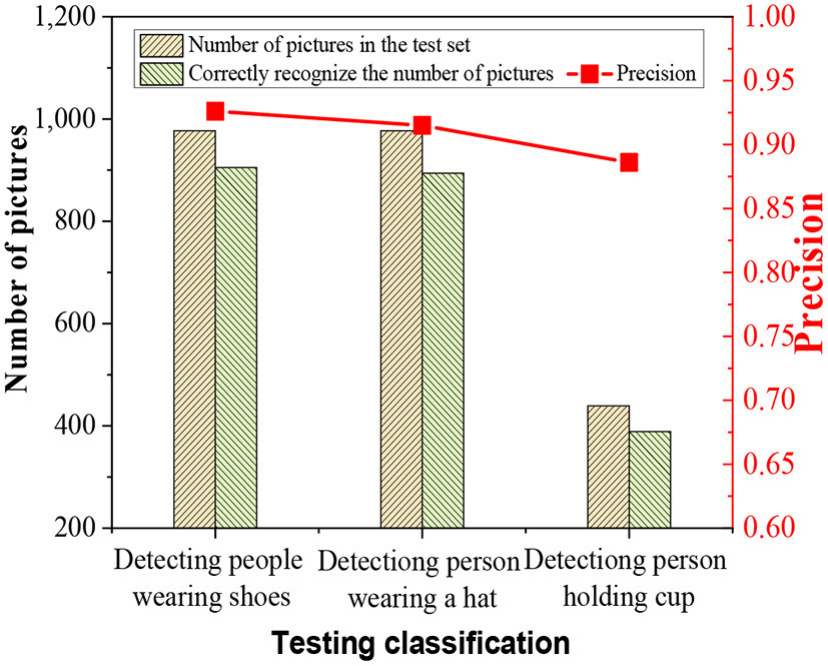
Behavior recognition result graph of the test set algorithm.

The performance of the designed algorithm to recognize human behavior is tested using the test set. The algorithm identifies the human skeleton information and target information in these behaviors, and Figure 7 shows the algorithm test results.

In Figure 7, the designed algorithm has a recognition accuracy of 0.93 for people wearing shoes and 0.91 for people wearing hats. The reason for missed detection of people wearing shoes is that the target position overlaps the position of the shoes when the person is facing the camera during detection. The position of the shoe can't be accurately identified, so the algorithm is unable to accurately recognize human behaviors. When the robot detects the situation of people wearing a hat, the human head is partially covered, and the hat is recognized, but the HBR is wrong. The recognition accuracy for a person holding a water glass is 0.88. The reason for low recognition accuracy may be that most of the water glasses are hidden by the palm. The water cup can't be accurately positioned, and the elbow and wrist block the water cup. The joint recognition algorithm can't locate the position of the hand joints, so the HBR algorithm can't accurately detect the behavior of a person holding a water cup.

### 4.2 Computation of operational complexity

**Figure 8:**
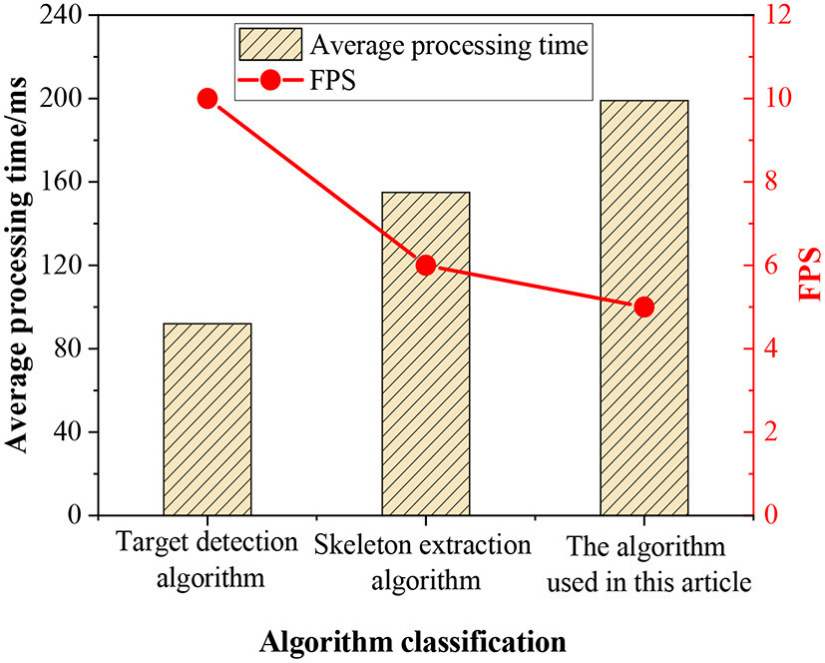
Comparison of algorithm efficiency.

The “openpose” is called through multiple threads to verify its time complexity. In the experiment, the target recognition algorithm and bone extraction algorithm are selected to recognize human behavior on the verification set. The processing results of the two algorithms are compared with the designed results, and the corresponding algorithm processing time and the number of images per second processed by each algorithm are recorded. Figure 8 shows the results of the target detection algorithm, bone extraction algorithm, and HBR algorithm adopted when they process the same data set.

As shown in Figure 8, although the time complexity of the HBR algorithm is higher than that of the target recognition algorithm and bone extraction algorithm, the algorithm focuses on both human behavior targets and bone information. Moreover, the algorithm also classifies and recognizes the behavior information contained in it. Therefore, when a single frame image is processed, the average processing time of the algorithm is 47 ms less than the total processing time of the target recognition algorithm and the bone extraction algorithm. Therefore, the algorithm adopted combines the characteristics of the target recognition algorithm and the bone extraction algorithm. Through multithreading, it reduces the image processing time and improves the detection efficiency of behavior recognition.

**Figure 9:**
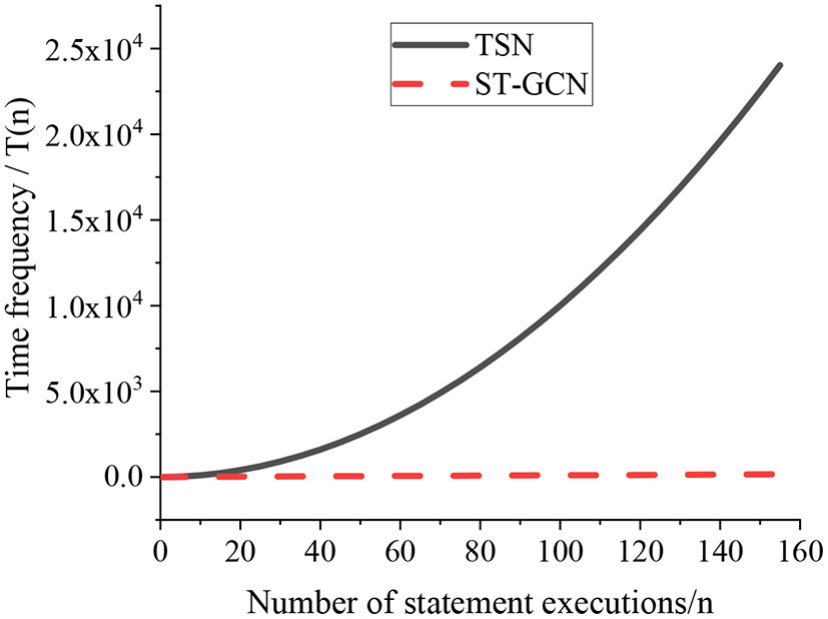
Time frequency comparison of different algorithms.

Time complexity is used to evaluate the time required for the algorithm to execute the program. After calculation of the time complexity, the degree to which the program uses the processor can be estimated. When an algorithm is designed, it is generally necessary to consider the system environment first, then weigh the time complexity and space complexity, and choose a balance point. Time frequency refers to the time it takes for an algorithm to execute. It can't be calculated theoretically, and it can only be known by running tests on the computer. However, since the time spent by an algorithm is directly proportional to the number of executions of the sentences in the algorithm, the time frequency can be counted, denoted as *T*(*n*), by calculating the number of executions of the sentences in the algorithm. Figure 9 shows the comparison result on the time frequency of the TSN algorithm and the ST-GCN algorithm.

As illustrated in Figure 9, the ST-GCN algorithm and the TSN algorithm show different time complexities when processing tasks. The comparison result shows that the greater the TSN algorithm is executed, the longer time it takes, and the execution time of the ST-GCN algorithm is relatively slow. Therefore, during the training and testing of the algorithm model, the execution time of the TSN algorithm is approximately equal to the calculation time of the designed model.

### 4.3 Performance comparison test

To verify the effectiveness of the designed algorithm, the ST-GCN, dual-stream CNN, and CNN algorithms are introduced for comparison with the designed algorithm on different data sets, as shown in Table 2.

**Table 2:** Performance comparison.

**Algorithm**	**Kinetics-skeleton data set**	**CGD2011**	**NTU-RGB + D data set**	**Data set in this work**
CNN	57.01%	60.7%	50.1%	49.87%
ST-GCN	74.45%	64.87%	63.23%	59.08%
Dual-stream CNN	74.76%	52.87%	84.46%	78.65%
The designed algorithm	83.77%	69.97%	81.50%	88.34%

As Table 2 shows, the best results presented by different models in different data sets are compared, and it is revealed that the algorithm designed in this work has advantages compared with similar algorithms. The recognition accuracy of the model designed in this work is above 70% in different experimental data sets, and the algorithm similar to this work has a better recognition effect on different data sets, but there are still significant differences in test results. This shows that the designed algorithm can better adapt to the changing experimental conditions to obtain better experimental results. The experimental results show that the algorithm designed in this work can identify human behavior more accurately by integrating the recognition results of the image sequence and video frame and then judge the consumer psychology of tourists by combining the classification of consumer psychological behavior described above.

## 5. Discussion

To judge the consumption psychology of tourists, consumers' behaviors are identified in this work. For example, when tourists try on shoes, they can judge their wearing and experience time as well as the wearable display actions to obtain their preference for shoes, guide them to visit the area of similar products, and enhance their consumption intention. Based on the above experimental results, it can be concluded that the combined behavior recognition algorithm based on CNN designed in this work is superior to the detection and recognition effect of a single algorithm for HBR and shows better recognition accuracy and excellent time complexity. Through the results of the model classification, it can evaluate the consumer psychology represented by their behaviors to judge the changes in their consumer psychology.

Therefore, if it is applied to the robot shopping guide service in shopping malls, the current behaviors of consumers can be quickly judged, and the relevant behavioral indicators can be combined with the consumption types of consumers to obtain the consumer psychological state. Compared with similar studies, the research in this work has the following outstanding contributions. First, the model designed in this work combines behavioral recognition with psychological judgment, which can judge the shopping intention of tourist destinations more effectively, stimulate the consumption intention of tourists in tourist destinations, and promote the development of the local economy. Second, this study employs deep learning technology to realize intelligent psychological situation analysis technology through automatic identification and analysis. Third, this study makes the research results more comprehensive and reasonable through the integration of psychological knowledge and science and technology and makes an important contribution to promoting the development of the consumer market. However, the constructed psychological behavior index system is not perfect because it can only simply judge the shopping state of consumers. Therefore, it is necessary to establish a complete index system in follow-up research so that the shopping guide robot can judge consumer psychology more accurately. In addition, the combination algorithm designed for the complex shopping environment of the mall is not perfect and needs to be improved constantly.

## 6. Conclusion

At present, social development has entered a high-speed stage, and promoting consumption has become an important means to promote social development. Therefore, to improve the consumer mood of users, a combined HBR algorithm is proposed by analyzing the relationship between tourism consumer psychology and residents' consumption behavior. It is applied to the tourist destination shopping guide robot to judge the shopping intention of consumers by identifying their shopping behavior and then obtain the type of consumers by relevant indicators to judge their consumer psychology. The experimental results show that the designed combination recognition algorithm has a good recognition effect. Compared with the single recognition algorithm, it can effectively reduce the image processing time and improve the detection efficiency of the algorithm. Compared with other algorithms, the proposed algorithm is more adaptable to the ever-changing data set to recognize human movements effectively. The innovation of this work lies in combining robot behavior recognition with current consumer psychology, classifying consumers by observing their shopping behavior, inferring their shopping psychology, and making personalized recommendations and services according to their shopping psychology characteristics. However, this work has some shortcomings. First, the performance and recognition accuracy of the design algorithm need to be further improved. Second, the relationship between consumer behavior and psychology needs to be further optimized. Finally, the research model is carried out under hypothetical experimental conditions, but the actual application environment is more complex, and the problems are also more complex. Therefore, the application scenarios of the model will be constantly updated in future studies to make the model adapt to more experimental backgrounds, thereby promoting the continuous development of the consumer market.

## Data availability statement

The raw data supporting the conclusions of this article will be made available by the authors, without undue reservation.

## Ethics statement

The studies involving human participants were reviewed and approved by Wuhan University of Science and Technology Ethics Committee. The patients/participants provided their written informed consent to participate in this study. Written informed consent was obtained from the individual(s) for the publication of any potentially identifiable images or data included in this article.

## Author contributions

All authors listed have made a substantial, direct, and intellectual contribution to the work and approved it for publication.
